# Effective Separation of Cancer‐Derived Exosomes in Biological Samples for Liquid Biopsy: Classic Strategies and Innovative Development

**DOI:** 10.1002/gch2.202100131

**Published:** 2022-05-09

**Authors:** Yujiao Xie, Xiawei Xu, Jie Lin, Yanping Xu, Jing Wang, Yong Ren, Aiguo Wu

**Affiliations:** ^1^ Cixi Institute of Biomedical Engineering International Cooperation Base of Biomedical Materials Technology and Application Chinese Academy of Science (CAS) Key Laboratory of Magnetic Materials and Devices and Zhejiang Engineering Research Center for Biomedical Materials Ningbo Institute of Materials Technology and Engineering CAS Ningbo 315201 P. R. China; ^2^ Advanced Energy Science and Technology Guangdong Laboratory Huizhou 516000 P. R. China; ^3^ Research Group for Fluids and Thermal Engineering University of Nottingham Ningbo China Ningbo 315100 China; ^4^ Department of Mechanical Materials and Manufacturing Engineering University of Nottingham Ningbo China Ningbo 315100 China; ^5^ Department of Electrical and Electronic Engineering University of Nottingham Ningbo China Ningbo 315100 China; ^6^ Key Laboratory of More Electric Aircraft Technology of Zhejiang Province University of Nottingham Ningbo China Ningbo 315100 China; ^7^ Nottingham Ningbo China Beacons of Excellence Research and Innovation Institute Ningbo 315040 China; ^8^ Key Laboratory of Carbonaceous Wastes Processing and Process Intensification Research of Zhejiang Province University of Nottingham Ningbo China Ningbo 315100 China

**Keywords:** cancers, cancer‐derived exosomes, liquid biopsies, separation methods

## Abstract

Liquid biopsy has remarkably facilitated clinical diagnosis and surveillance of cancer via employing a non‐invasive way to detect cancer‐derived components, such as circulating tumor DNA and circulating tumor cells from biological fluid samples. The cancer‐derived exosomes, which are nano‐sized vesicles secreted by cancer cells have been investigated in liquid biopsy as their important roles in intracellular communication and disease development have been revealed. Given the challenges posed by the complicated humoral microenvironment, which contains a variety of different cells and macromolecular substances in addition to the exosomes, it has attracted a large amount of attention to effectively isolate exosomes from collected samples. In this review, the authors aim to analyze classic strategies for separation of cancer‐derived exosomes, giving an extensive discussion of advantages and limitations of these methods. Furthermore, the innovative multi‐strategy methods to realize efficient isolation of cancer‐derived exosomes in practical applications are also presented. Additionally, the possible development trends of exosome separation in to the future is discussed in this review.

## Graphical abstract

This paper provides an overview of the latest advances in separation of cancer‐derived exosomes with a discussion of the advantages and limitations of each method. The work highlights the importance of combining microfluidics with multiple separation strategies to develop integrated microsystems for rapid separation of exosomes with high stability and sensitivity, which will ultimately enable a facile and effective operation for early diagnosis and cancer treatment.

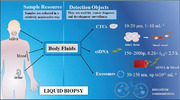

## 1 Introduction

**Figure 1:**
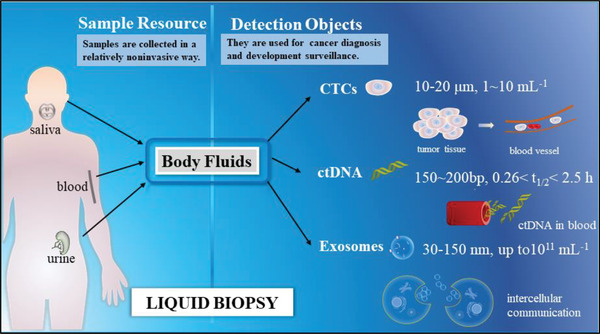
Sample resource and detection targets of liquid biopsy. Biological samples for liquid biopsy are generally collected from blood, urine, and saliva. CTCs and ctDNA are two commonly used targets of liquid biopsy, but they have the shortcomings of rarity and instability, respectively. In contrast, exosomes involved in intercellular communication are relatively stable and rich.

Cancer, also known as malignant tumor, has become a major threat to human life throughout the world on account of its high incidence and mortality rate. As the global cancer data released by International Agency for Research on Cancer, around 19 million new cancer cases had been confirmed in 2020, and this number would predictably rise to more than 32 million over the next 20 years.^[^ ^1^ ^]^ According to the worldwide cancer analysis, one out of every 5 men or women is likely to fight against cancer during their lifetime, while 1 in 8 men or 1 in 11 women will die from cancer.^[^ ^2^ ^]^ In response to such severe challenges, universal efforts have been invested not only into exploring clinically effective anti‐cancer drugs but also in developing reliable methods to perform diagnosis and surveillance of cancer. The tissue biopsy is an important tool for cancer diagnosis and tissue samples are directly obtained from patients’ tumor sites and further treated for pathological testing. It has been the gold standard for cancer diagnosis for a long time. However, this method has shown some limitations in clinical practice. First, the operation process is invasive and sophisticated, which may exert great pain on most patients.^[^ ^3^ ^]^ In addition, due to the tumor heterogeneity, the samples obtained by tissue biopsy may not effectively describe the overall characteristics of tumor. These disadvantages apparently limit the accuracy of cancer detection and increase the difficulty in dynamic detection of tumor.^[^ ^4^ ^]^ To facilitate the detection of cancer, another novel method, liquid biopsy, has been extensively developed. This approach aims to obtain and analyze disease information from cancer‐derived components, mainly including circulating tumor cells (CTCs), circulating tumor DNA (ctDNA), and exosomes. The sample sources are body fluids, including blood, urine, and saliva, and they are collected through a relatively non‐invasive way, such as blood draw or urine collection, which could highly reduce the suffering of patients.^[^ ^5^ ^]^ Besides, it also has been reported that liquid biopsy can present the entire genomic landscape of the tumor and the problem of tumor heterogeneity can be overcome.^[^ ^6^ ^]^ More importantly, liquid biopsy is a repeated test in a long term, which provides a convenient method for monitoring dynamic change and therapeutic effects of tumor.^[^ ^7^ ^]^ **Figure** **1** illustrates common sample resource and detection targets of liquid biopsy.

Ashworth first proposed the concept of CTCs in 1968, and it was later defined as tumor cells that spread from primary site into the peripheral blood. CTCs have complete cellular structure and are considered to be the main biological basis for hematogenous metastasis of malignant tumor.^[^ ^8^ ^]^ The number of CTCs is closely related to the prognosis of patients, which has been demonstrated in post‐treatment monitoring of various cancers, such as breast cancer,^[^ ^9^ ^]^ prostate cancer,^[^ ^10^ ^]^ and colon cancer.^[^ ^11^ ^]^ However, on account of the rarity and heterogeneity of CTCs in circulation, the application of CTCs remains a great challenge for molecular analysis and cancer diagnosis.^[^ ^12^ ^]^

In 1948, Mandel and Metais first reported the existence of circulating free DNA (cfDNA).^[^ ^13^ ^]^ After decades, researchers discovered that the number of cfDNA were related to the development of tumors and then ctDNA was successfully confirmed and sequenced. In recent years, ctDNA has been found to have important application value in the early detection of early non‐small cell lung cancer^[^ ^14^ ^]^ and dynamic monitoring of breast cancer,^[^ ^15^ ^]^ ovarian cancer,^[^ ^16^ ^]^ and esophageal cancer.^[^ ^17^ ^]^ However, the amount of ctDNA in the early stage of tumor remains very small and the half‐life time is quite short (0.26 < *t*_1/2_ < 2.5 h). In addition, the extraction and sequencing process of ctDNA costs much money and time, which greatly restricted its applicability.

Exosome is a type of extracellular vesicles (EVs) secreted by parent cells and equipped with complete biological membrane, containing various protein and nucleic acid. It was first discovered during reticulocyte maturation by Johnstone in 1987.^[^ ^18^ ^]^ For a long time, exosomes had been only considered as transport vehicles carrying cell waste, and only a few studies on exosomes had been conducted. The role of exosomes in immune response, intercellular communication, and transmission of genetic information had not been recognized until the end of the 20th century. The 2013 Nobel Prize in Physiology and Medicine was then awarded to three scientists (Prof. James E. Rothman, Randy W. Schekman, and Thomas C. Südhof) who had elucidated the regulation mechanism of exosomes in cell, including i) necessary genes involved in vesicle transport, ii) the protein operation mechanism of the fusion of vesicle and target cell to deliver information, and iii) the mechanism of the signaling system to accurately direct the vesicle to release biological molecules.^[^ ^19^ ^]^ There are two types of EVs that have been recognized. One is microvesicles (MVs) that are directly secreted from the cell membrane by “budding” with particle size of 100–1000 nm. The other type of EVs is exosome (30–150 nm, up to 10^11^ mL^−1^), which originates from endocytic vesicles and spills through fusion with cell membrane after being released by early endosomes and multivesiclar bodies (MVB). After being secreted by parent cells, exosomes utilize their transmembrane proteins or lipid ligands to exert pleiotropic biological activities with the corresponding receptors on other cells, and then transfer cytoplasmic proteins and nucleic acids to recipients via membrane fusion.^[^ ^20^ ^]^ Based on this, exosome has been consecutively reported to play a crucial role in the occurrence and development of cancer, including tumor progression, metastasis, and facilitating immune escape.^[^ ^21^, ^22^, ^23^ ^]^ In the study on the biological diagnosis of early pancreatic cancer, biologists successfully identified glypican‐1 (GPC1) as a proteoglycan, which was specifically enriched on the surface of cancer‐derived exosomes. The flow cytometry has been further used to monitor and isolate GPC1‐positive exosomes in the serum of pancreatic cancer patients and mice, and it confirms that such exosomes have high specificity and sensitivity, which can distinguish healthy individuals from early and late pancreatic cancer patients.^[^ ^24^ ^]^ Similarly, the concept of separating and detecting tumor‐derived exosomes as an important target for liquid biopsy in early diagnosis via different methods to distinguish cancer patients from healthy groups has also been realized in many other cancers, such as breast cancer,^[^ ^25^ ^]^ liver cancer,^[^ ^26^ ^]^ prostate cancer,^[^ ^27^ ^]^ and ovarian cancer.^[^ ^28^ ^]^

Making full use of the exosomes in cancer diagnosis and treatment involves two indispensable steps: i) effective separation of the exosomes from biological samples and ii) accurate analysis of their protein and nucleic acid contents by downstream analysis, like western blotting and polymerase chain reaction.

At present, the most commonly used source in clinic for liquid biopsy of cancer is blood. It is well known that composition of blood is complex and varied, since apart from EVs, there also exist a large number of different cells such as thrombocyte, hemameba, and erythrocyte, and many macromolecular substances such as proteins and nucleic acids, and these interferents usually show different properties in physical and biological aspects from exosomes. It is quite clear that only when exosomes are effectively extracted from samples can subsequent downstream analysis be performed and disease information be presented. Thus, it has become an important research field and attracted lots of efforts to remove undesired substances and enrich targeting exosomes.

A panoramic view of the current research status of exosomes reveals two mainstream strategies which have gradually formed in the field of exosomes isolation and detection. One is to screen out total exosomes based on their physical characteristics, such as the density and size, with the help of centrifugal force, gravity, and applied fields. The contained proteins and nucleic acid can be subsequently extracted for downstream molecular analysis to compare the differences among the samples;^[^ ^29^ ^]^ the second is to utilize affinity principle to specifically capture the required exosomes via special recognition between protein markers on the surface of exosomes and their corresponding antibodies/aptamers. Finally, these specially selected exosomes can be detected and analyzed to obtain cancer‐related information.^[^ ^28^ ^]^ Currently, a number of review papers have analyzed and summarized the separation strategies of exosomes. Most of them have clarified the principles underlying the exosome separation methods and compared their advantages and disadvantages. However, few articles have concentrated on the isolation of cancer‐derived exosomes, and the application of these exosomes in liquid cancer biopsies has rarely been mentioned.

This review aims to summarize the strategies of separation exosomes according to their physical properties and demonstrate the emerging microfluidic methods developed in recent years according to the design of micro‐structure and addition of external fields. We will explain immunoaffinity‐based separation approach and highlight some integrated multi‐strategy methods. Finally, the conclusion and outlook are presented.

## 2 Methods of Exosome Separation

According to the physical properties of exosomes (such as density, size, solubility) and biological properties (immunoaffinity), many separation methods have been proposed (**Table** **1**). The conventional separation methods laid the foundation for the research and application of the pathogenic mechanism of exosomes, and established the gold standard for the separation of exosomes. However, these methods are only suitable for laboratory research, since the operation steps are time‐consuming and usually consume a relatively large volume of liquid samples (a few milliliters of blood or hundreds of milliliters of cell culture fluid). In order to save time and effort in the detection of tumor‐derived exosomes, emerging microfluidic techniques used to efficiently isolate exosomes have been gradually developed over the recent years.^[^ ^30^ ^]^ On the microfluidic platform for exosome separation, the collected liquid samples are processed in two basic patterns. One is to spread sample solution in different channels comprised of various specially designed microstructures, and then biological particles can be distinguished according to their different flow trajectories. The other is to separate particles in the sample based on their propensity to move in different directions under the action of an external field. Such a platform is also called a laboratory on a chip, namely lab‐on‐chip. As a result, not only many laboratory achievements have been published, some commercial products also have been developed and issued by technology companies. This section will clarify the theoretical basis of exosome separation and give examples of traditional and emerging microfluidic methods.

**Table 1:** A brief summary of different conventional exosome separation methods

Principle	Method	Advantage	Disadvantage
Density	Differential centrifuge	Easy scaling up	Mechanical damage Time‐consuming Labor‐intensive
	Density gradient centrifugation	Increased purity compared to differential centrifuge	Mechanical damage, cumbersome workload
Solubility	Precipitation	Ease of use time‐saving	Protein and polymer contamination
Size	Membrane Separation	High purity	Limited volume
	Size‐exclusion chromatography	Easy to handle	Protein contamination
	Microarray‐based separation	High resolution	Limited throughput pillar clogging
	Active Separation in microchannel	High recovery rate	Potential structural damage analog interference
Immunoaffinity effect	Antibodies separation	High specificity	High cost
	Aptamer separation	Easy scaling up high specificity	Ease of degrading
Multiple strategy	Integrated microfluidic chips	Automation time‐saving	Complex design process sophisticated fabrication

### 2.1 Methods Based on Physical Properties to Collect Total Exosomes

#### 2.1.1 Traditional Methods Based on Density of Exosomes

**Figure 2:**
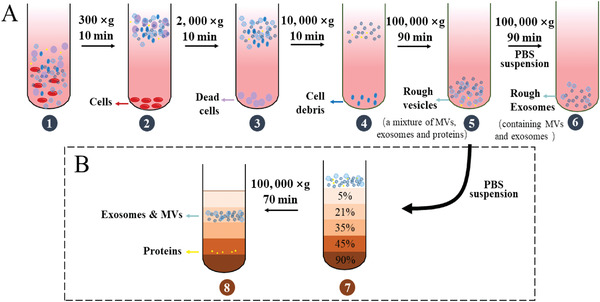
Diagram of differential centrifugation and density gradient centrifugation. A) Differential centrifugation, under a series of increased centrifugal conditions, cells, dead cells, cell debris, and proteins are gradually removed to obtain rough exosomes; B) Density gradient centrifugation, the PBS suspension containing MVs, exosomes, and proteins is put on the top layer of gradient solution, rough exosomes and proteins can be distributed in different regions.

The most classic method currently used is the differential centrifugation method followed by ultracentrifugation, which refers to the separation of the required components in the mixture under a series of gradually increasing centrifugal acceleration conditions and removal of other unnecessary components. The basic principle is that the density of substances in mixed solution varies depending on their shape, size, and mass. Generally, different densities imply different sedimentation speeds under the effect of centrifugal force. Thus, substances can be gradually separated via using a series of centrifugation. In the practical separation of exosomes (**Figure** **2**A), low‐speed centrifugation (300–2000 × *g*, 20 min) is employed first to remove cells and dead cells in the biological sample. Then cell debris is precipitated under centrifugation at 10 000 for 10 min. After that, centrifugation at a higher speed (100 000 × *g*, 90 min) is subsequently introduced to obtain rough vesicles, which contain MVs, exosomes, and protein aggregate. Finally, this mixture is resuspended in PBS and rough exosomes containing MVs are collected after the last step of high‐speed centrifugation (100 000 × *g*, 90 min). The experimental design of this method is not complicated and does not involve sophisticated sample processing procedures, so it has the advantage of easy scale‐up and can handle samples with volumes up to several hundred milliliters. Therefore, it has been widely used for isolation of cancer‐derived exosomes from several liquid sample sources, including blood,^[^ ^31^ ^]^ cell culture supernatant,^[^ ^32^ ^]^ and urine.^[^ ^33^ ^]^ However, the shortcomings are also obvious. The entire process relies heavily on cumbersome centrifugal equipment, and is quite time‐consuming and labor‐intensive. In addition, the recovery rate^[^ ^34^ ^]^ and purity^[^ ^35^ ^]^ of the final product obtained by differential centrifugation are limited because the product is a mixture of MVs, exosomes, and other non‐vesicle components (such as apoptosis bodies and some protein aggregates) which have similar density with EVs. This result may compromise the accuracy and reliability of exosome‐based diagnosis and therapy.^[^ ^36^ ^]^

In order to increase the purity of the differential centrifugation method, researchers introduced sucrose or iodixanol solution in the last step of the above ultracentrifugation. This method, called density gradient centrifugation, is to place the PBS suspension containing rough vesicles on the top layer of the prepared 5–90% gradient solution, and after a certain period of high‐speed centrifugation (such as 100 000 × *g*, 70 min^[^ ^37^ ^]^), the rough exosomes and other remaining impurities (such as protein aggregates) can be distributed in different regions of density (Figure 1B). Compared with the differential centrifugation method, this approach has made some progress in the purity of exosomes.^[^ ^38^ ^]^ But it cannot be ignored that the long processing time will not only increase the total cost of separation, but also may impair the maintenance of morphology and biological activity of exosomes.

Because these two types of EVs, exosomes, and MVs have overlap in size and density, although they show different origins, it is still difficult to distinguish them from each other completely.^[^ ^39^, ^40^ ^]^ Therefore, despite the method of density‐based exosomes separation being easy to use, it suffers from the shortcomings of limited purity and cumbersome workload.

#### 2.1.2 Methods Based on Solubility of Exosomes

**Figure 3:**
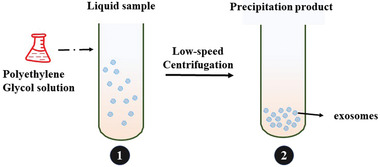
Diagram of precipitation method. Polyethylene glycol solution is added into collected sample and precipitated exosomes can be obtained after incubation and low‐speed centrifugation.

Similar to the structure of cell membranes, exosomes can stably exist in aqueous solutions due to the hydrophilic phosphorus ends and membrane proteins on their surfaces. However, when a highly hydrophilic polymer is introduced into the exosome solution, it will interact with the water molecules surrounding the exosomes and form a hydrophobic microenvironment, resulting in a decrease in the solubility of exosomes. Thus, exosomes can be precipitated in the sample due to lack of water molecules. Based on this principle, researchers added polyethylene glycol solution to a biological sample which had been initially purified removing cell debris, and incubated the resulting mixed solution overnight at a low temperature (**Figure** **3**). After twice low‐speed centrifugation (5000 × *g*), the required exosomes can be collected.^[^ ^41^ ^]^ This method is easy to operate with simplified purification process, which highly facilitates its popularization and scale‐up.

At this stage, exosome purification kits based on hydrophilic polymer precipitation have been put on the market in many countries, such as Total Exosome Isolation Reagent developed by Invitrogen and ExoQuick by System Biosciences of the United State, Exosome Isolation Kit by Exiqon of Denmark, Exosome Purification Kit from Norgen Biotek of Canada and GS Exosome Isolation Reagent developed by Geneseed Biotech of China.^[^ ^36^ ^]^ Among them, ExoQuick is prominently widely accepted, and has played a significant role in various cancer research, such as ovarian cancer,^[^ ^42^ ^]^ breast cancer,^[^ ^43^ ^]^ gastric cancer,^[^ ^44^ ^]^ colon cancer,^[^ ^45^ ^]^ and liver cancer.^[^ ^46^ ^]^ Even so, the precipitation method still has some shortcomings. First, while the polymer reduces the solubility of exosomes, it also co‐precipitates some other protein aggregates, so that there are excess proteins in the final product that cannot be removed, such as albumin and immune globulin.^[^ ^47^ ^]^ Otherwise, it is worth mentioning that the content of RNA (mRNA and miRNA) extracted from exosomes achieved by this method is also reported significantly higher than that of ultracentrifugation, indicating that this method is highly suitable for the nucleic acid research.^[^ ^48^ ^]^ In addition, except the protein contamination, residual polymers also exist in the final system of isolated exosome solution and these impurities are reported to have unexpected cell cytotoxicity and can adversely affect downstream analysis.^[^ ^49^ ^]^ Therefore, even if this method has been widely used, it still needs to pay a lot of effort to remove undesired impurities.

#### 2.1.3 Methods Based on Size of Exosomes

The diameter of most exosomes is tens of nanometers, which is the material basis of exosome separation methods based on size. The design principle of this method is that when the sample flows through some constructed pores, channels, or arrays, the motion trajectories of the micron‐sized particles (cells), the macromolecular substances (nucleic acids and proteins), and exosomes tend to move into different directions. During the separation process, there are passive methods relying only on gravity field without external force, and active methods by introduction of external force, like electrical, sound, and thermal field. Generally speaking, the former is easy to operate, while it is time‐consuming and the purity is limited. In comparison, the latter requires precise control and sophisticated device, but the isolation of exosomes is quite efficient.

##### Membrane Separation

**Figure 4:**
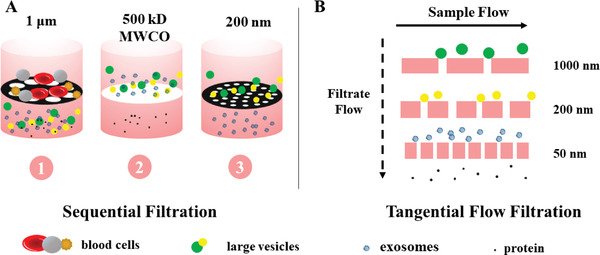
Diagram of sequential filtration and tangential flow filtration. A) Sequential filtration to separate exosomes. The blood sample is sequentially passed through a 1000 nm filter, a 500 Kd MWCD membrane, and a 20 nm filter to remove blood cells, large vesicles, and proteins, respectively; B) Tangential flow filtration to separate exosomes. The liquid is kept flowing in a direction parallel to the membrane under constant pressure, so that the clogging issue can be overcome.

Membrane filtration is also a traditional separation method, which refers to the use of a membrane with designed microporous structure of a specific size to separate particles in the liquid according to their size. For the separation of exosomes, a micron‐scale filter is usually used to remove cells and cell debris, and a nano‐scale filter is combined to remove large vesicles and obtain exosomes. The relatively popular method for exosome separation is called sequential filtering mode.^[^ ^36^ ^]^ As shown in **Figure** **4**A, the blood sample is first passed through a 1000 nm filter to remove some large particles, and then the filtrate is flowed across a second filter of 500‐kD MWCO ultrafiltration membrane to remove free proteins and other small particles. At last, a 200 nm filter is applied to collect exosomes with a diameter between 50–200 nm. Based on this fine sequential filtration method, an ExoMir exosome isolation kit has been developed and released to market by Bio Scientific Corporation.^[^ ^50^ ^]^ Compared with the differential centrifugation method, this method has significant advantages, including saving lots of time and avoiding the possibility of exosomes being damaged by shear force. But it also has the disadvantage that high‐concentration liquid samples often get clogged when flowing through the filter membrane. Membrane clogging issue will not only reduce the service life of the membrane, but also impair the separation efficiency.^[^ ^51^ ^]^

To address this issue, tangential flow was introduced into the filtration system. It means that the liquid to be filtered is kept flowing in a direction parallel to the membrane under constant pressure (usually produced by pump), while the particles move in the direction tangential to the membrane.^[^ ^52^ ^]^ Compared with the traditional dead‐end filtration, this tangential flow filtration (TFF) protocol (Figure 4B) can effectively reduce the possibility of cells clogging in the membrane pores and obtain a high recovery rate.^[^ ^53^ ^]^ In the early days, it was used for the separation of fabricated nanoparticles,^[^ ^54^ ^]^ and later it has also been applied for the separation of exosomes in urine.^[^ ^55^ ^]^

**Figure 5:**
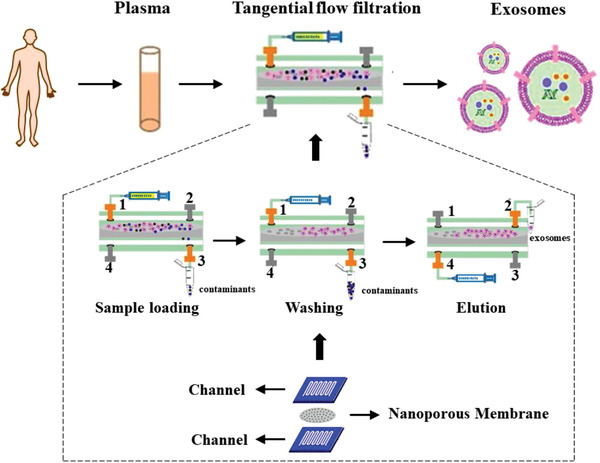
Exosome isolation on a chip using tangential flow filtration. After being injected in a serpentine channel, exosomes in pre‐treated sample are captured on a membrane with a pore size of 100 nm, while proteins some small particles are washed out. Reproduced with permission.^[^ ^56^ ^]^ Copyright 2021, Elsevier.

Recently, Han^[^ ^56^ ^]^ successfully combined TFF with microfluidic technology, and transferred the process of exosomes isolation by filtration to a micron‐scale chip (**Figure** **5**). The sample that has been pretreated under high‐speed centrifugation (10000 × *g*, 30 min) to remove cell debris is injected into a serpentine channel with a width of 500 µm from the upper inlet 1. Exosomes are captured on a porous membrane with a pore size of 100 nm, while proteins and some small particles are washed out at outlet 3. After twice rinse with deionized water, the inlet 1 and outlet 3 are closed, and deionized water is injected from the bottom inlet 4 to elute the exosomes through outlet 2. The results showed that the cervical cancer cell exosomes obtained by this method are more uniform in particle size than those obtained by differential centrifugation. In addition, it should be noted that the intensity of filtration pressure needs to be carefully controlled,^[^ ^57^ ^]^ otherwise it may cause irreversible damage to the complete morphology of the exosomes.

##### Size‐Exclusion Chromatography (SEC) Method

**Figure 6:**
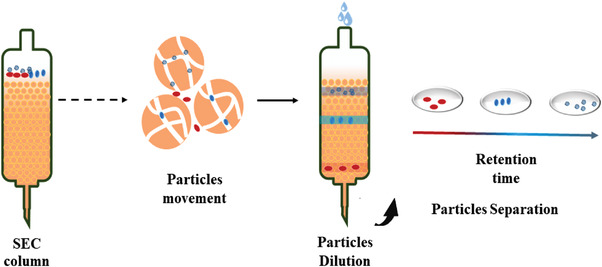
Diagram of SEC. Particles with different size in the biological sample can be separated according to the difference in the retention time flowing through the SEC column.

In the field of compound analysis, to separate components with different molecular weights or sizes, in addition to separation by membrane method, some tightly packed stationary phases can also be used. The stationary phases are usually a resin material with a porous structure or a hydrophilic polymer (such as dextran, agarose) that can form a gel and they are stuffed in a column allowing sample fluid and eluent to flow. This system is defined as SEC, which aims to separate components according to the difference in particles’ flow distance. When the sample flows through stationary phase, large‐sized cells cannot enter the pores of stuffed material and can only move forward onto the path around the stationary material. On the contrary, exosomes with smaller shape can penetrate into the pores and flow through most of pores. Therefore, the moving distance of exosomes is significantly increased, and it takes longer time to elute out the exosomes. Various components in the biological samples can be separated according to the difference in the retention time of substances shifting in the stationary phase (**Figure** **6**). SEC has been also developed rapidly. At present, qEV (iZON) and PURE‐EVs are already released on the market, and mini‐SEC columns are used to separate cancer‐derived exosomes in worldwide laboratories. Hong^[^ ^58^ ^]^ obtained exosomes with complete structure and excellent functional activity from plasma samples of patients with acute myeloid leukemia and head and neck squamous cell carcinoma (HNSCC) within 30 min via using mini‐SEC, and pointed out that these advantages were of great significance of downstream analysis. Ludwig^[^ ^59^ ^]^ creatively optimized in vitro cell culture conditions by using the recovery and purity information of exosomes obtained by mini‐SEC. Even so, this method is still reported that there may be some protein contamination problems, because the exosomes obtained by SEC showed a larger distribution width in a small diameter range. Therefore, some scholars have proposed modified strategies combined with ultrafiltration membrane method to remove excess impurities, which can maintain the functional integrity of exosomes and improve purity.^[^ ^60^ ^]^

##### Microarray‐Based Separation

**Figure 7:**
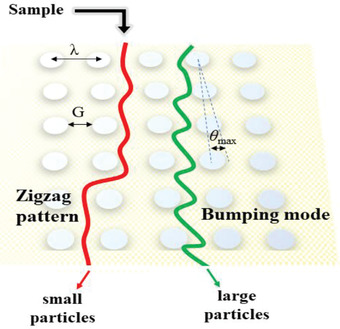
Diagram of DLD. After sample is passed through the array, small particles move in a zigzag pattern, while large particles flow out the array in a bumping mode.

Inspired by the different trajectories of particles in SEC, researchers subsequently proposed some emerging microarray‐based structures for the separation of exosomes. Generally, particles with different sizes will show different trajectories in the specially designed microarray device. Some particles will move forward in a zigzag pattern when flowing in a specially designed parallel array, while others will flow out of the array in the form of bump. This difference is called deterministic lateral displacement (DLD).^[^ ^61^ ^]^ As can be seen from **Figure** **7**, identical micropillars are arranged in parallel, but the micropillars of each column are offset from the previous row by a regular distance in the next row. In practical condition, after the sample enters the array, small particles will follow the initial streamline and move in a zigzag trajectory, while the large particles will bump with the pillars and move laterally to the next streamline as a bumping mode until flowing out of the array area. The cut‐off size parameter of the two moving modes is called the DLD critical diameter *D*_c_, and this value is associated by the geometry of the array, which is affected by some important parameters, pillar gap *G*, pillar pitch λ of and the angle between the first row and the last row of micro‐pillars θ_max_. According to this principle, Smith^[^ ^62^ ^]^ reported a nanoDLD chip and successfully applied it into the separation and enrichment of exosomes in serum and urine, and proved that this method can increase by about 50% on the basis of differential centrifugation and SEC when using the same small sample volume. In addition, after injecting serum form prostate cancer patient into this system, nucleic acid sequencing of the isolated product also verified the ability of nucleic acid to characterize the aggressiveness of prostate cancer.

##### Active Separation in Microchannel

Membrane method, SEC, and DLD are all based on the difference in particle trajectories, for which it is necessary to construct various barriers in device, such as porous channels and microarrays. Thus, these approaches can be considered as passive strategies, because they highly rely on the nature of exosomes themselves. In recent years, it has been demonstrated that the uneven forces exerted on biological particles in the physical field can also lead to deflected motions of the particles, thus giving rise to many new separation methods. In terms of exosome separation, there are three commonly used external force fields, namely dielectrophoresis (DEP), acoustic radiation, and centrifugal force. The principles and applications involved in these three forces will be explained below.

**Figure 8:**
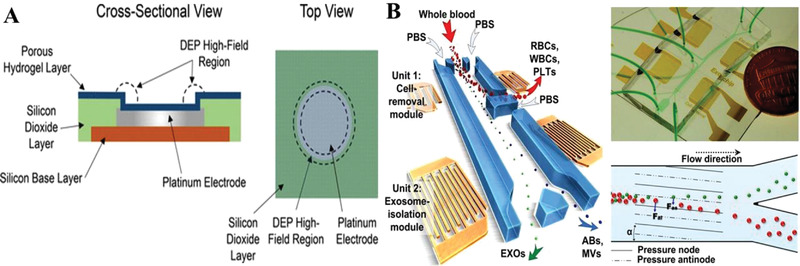
Diagram of Active separation in microchannel under DEP force and acoustic radiation force. A) An electric microarray chip based on DEP technology, which can quickly separate and recover the exosome of glioblastoma from undiluted blood samples. Reproduced with permission.^[^ ^63^ ^]^ Copyright 2017, American Chemical Society. B) The device is divided into microcellular removal module and exosome separation module. Reproduced with permission.^[^ ^65^ ^]^ Copyright 2017, National Academy of Sciences.

DEP force is a kind of force that allows polarized particles in non‐uniform electric field to perform directional migration. The value of DEP force depends on the size of suspended particles and is related to the electrical properties of media. Generally, by applying alternating voltage on a microelectrode in solution, DEP phenomenon can be created and DEP force thus is applied in manipulation of micro and nano particles. Ibsen^[^ ^63^ ^]^ proposed an electric microarray chip based on DEP technology, which can quickly separate and recover the exosome of glioblastoma from undiluted blood samples (**Figure** **8**A). The separation principle of the chip is that according to the different dielectric properties of exosomes and other components in plasma, exosomes are attracted to the edge region of microelectrode, that is, the strong field region of dielectric electrophoresis, while cells and macromolecular proteins are drawn into the weak field of electrophoresis. Besides, Sonnenberg^[^ ^64^ ^]^ successfully separated DNA and other nanoscale substances from blood by similar microarray electrode methods. By using this method, the exosome recovery and purity were high, and the required plasma sample volumes were small. However, one of the potential disadvantages of this method is that the exosome may be damaged by electrochemical phenomena resulted from direct contact between exosome and electrode.

By skillfully combining microfluidic technology and acoustics to form acoustic fluid (Acoustofluidics), researchers can effectively realize the sorting and manipulation of particles. The principle is that particles of different sizes are subjected to differential acoustic radiation force and viscous force in microfluidic sound field. The viscous force is proportional to the radius of the particle, while the acoustic radiation force is proportional to the volume of the particle. For larger particles, the acoustic radiation force plays a dominant role and the particles move to the acoustic node; while for the smaller particles, the viscous force counteracts most of the acoustic radiation force and the lateral motion of the particles is weak. Under the combined action of acoustic radiation force and viscous force, particles of different sizes will move to different exits, and then the separation of particles will be realized. Although acoustofluidics technology is widely used in cell manipulation and isolation, but the original acoustic fluidic device is only suitable for the separation of two types of particles present in system. Therefore, it is difficult to separate exosomes from complex blood. In response to this deficiency, Wu^[^ ^65^ ^]^ developed a new type of acoustic fluid device, in which exosomes can be separated quickly and effectively from whole blood samples without labeling and contact. As shown in Figure 8B, the device is divided into microcellular removal module and exosome separation module. First, A lower frequency (19.6 MHz) acoustic wave is used in the microcellular removal module to remove the larger red blood cells, white blood cells, and platelets; A higher frequency (39.4 MHz) sound wave is used in the exosome isolation module to separate the exosome from the components of the EVs (apoptotic bodies, larger EVs, etc.). Lee^[^ ^66^ ^]^ also demonstrated an exosome separation method based on an acoustic nanofiltration system. The method is based on differences in the size and density of EVs and other components. Nanoscale vesicles (<200 nm) can be isolated from cell culture medium and erythrocyte products by ultrasonic standing wave. The exosome separation method based on acoustic fluid has the advantages of good biological characteristics, good purity, and satisfactory recovery rate. It is a novel separation method. However, the separation principle is based on the objects’ size and acoustic impedance characteristics, so it is inevitable to be disturbed by other components in plasma which are similar to the exosome in size and acoustic impedance characteristics.

The centrifugal microfluidic platforms provide another mode of separation by integrating multiplexing network of microchannels and chambers in a circular‐shaped platforms or compact discs (CD), and they have been widely used to realize inexpensive, disposable, and high through‐put cell separation, which are easy to handle and do not need sophisticated equipment.^[^ ^67^ ^]^ On the centrifugal microfluidic platform, the flow/plasma will be activated by centrifugal force, and the magnitude of centrifugal acceleration is very large. For example, at the speed of 2000 r min^−1^ and the centrifugal radius of 20 mm, the centrifugal acceleration can reach 876.4 m s^−2^, which is about 89 times the acceleration of gravity.^[^ ^68^ ^]^ Such great force and acceleration facilitate movement of blood cells or other particles toward to bottom of a chamber on the centrifugal disc, effectively achieving the goal of cell removal. It is worth mentioning that the application of this platform has successfully promoted the development of CTCs separation methods.^[^ ^69^ ^]^ In practical applications, cell separation on a centrifugal microfluidic chip serves as a platform for pretreatment of blood for exosome separation. The role of this method is mainly to remove cells from complex biological samples, but it needs to be combined with other strategies in order to obtain exosomes with high purity or specificity.

##### Other Separation Approaches

**Figure 9:**
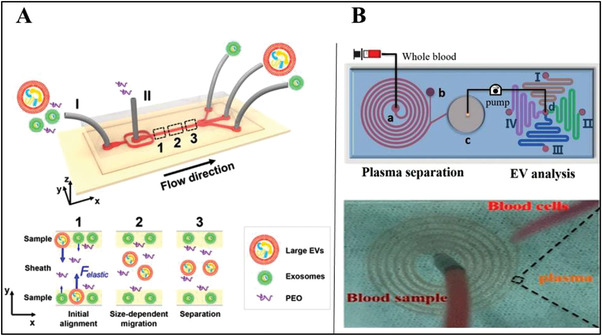
Diagram of separation of exosomes based on viscoelastic microfluidics and inertial microfluidics. A) The larger EVs are moved to the center of the channel by a larger viscoelastic force, while the smaller exosome has limited viscoelasticity and less displacement. Reproduced with permission.^[^ ^70^ ^]^ Copyright 2017, American Chemical Society. B) A six‐loop spiral device on a chip to separate blood cells from the whole blood of ovarian cancer patients. Reproduced with permission.^[^ ^28^ ^]^ Copyright 2020, American Chemical Society.

In viscoelastic microfluidics, a viscous elastic force can act on a particle and make it move into different direction. The magnitude of the viscoelastic force and its displacement is related to the size of the particle. In contrast to other techniques such as acoustic fluid and DEP, the viscoelastic separation can accurately manipulate particles without additional field force by using the microfluidic viscoelastic separation method. Therefore, the viscoelastic force of microfluidics can be used as a simple, label‐free technique for particle manipulation and separation. Liu^[^ ^70^ ^]^ proposed a method that uses polyethylene oxide polymers (Polyoxyethylene, PEO) as a medium additive to change the viscoelasticity of the fluid. As shown in **Figure** **9**A, in the process of separation, controlling the flow rate of the inlet and sheath makes the sample starts on both sides of the microchannel. The larger EVs are moved to the center of the channel by a larger viscoelastic force, while the smaller exosome has limited viscoelasticity and less displacement. At the end of channel, exosomes and larger EVs can move to different exits.

Similar to the centrifugal microfluidic platform, there is another sample pre‐processing strategy used for exosome separation, namely the inertial microfluidics in the spiral ring. According to the principle of inertial microfluidics, the flow state of the particles in the spiral channel is determined by the relative magnitude of the inertial lift force *F*_L_ and the Dean drag force *F*_D_. The ratio of the two forces is closely related to the radius of curvature and hydraulic diameter of the channel and the size of the particles. When the inertial lifting force dominates, it can push micron‐scale particles quickly move to the equilibrium position and form an inertial focus flow. Therefore, by designing a spiral ring with specific parameters, cells in sample can be screened out and the centrifugal process with shear damage can be avoided. Zhou^[^ ^28^ ^]^ designed a six‐loop spiral device on a chip to separate blood cells from the whole blood of ovarian cancer patients and sent the product to the subsequent module of specific exosome capture (Figure 9B). This spiral ring has a total length of 23 cm, a width of 500 µm, and a height of 50 µm, which can achieve the separation efficiency of nearly 100% for blood with a hematocrit of 0.5% and 1%.

### 2.2 Methods Based on Biological Characteristics of Exosomes

**Figure 10:**
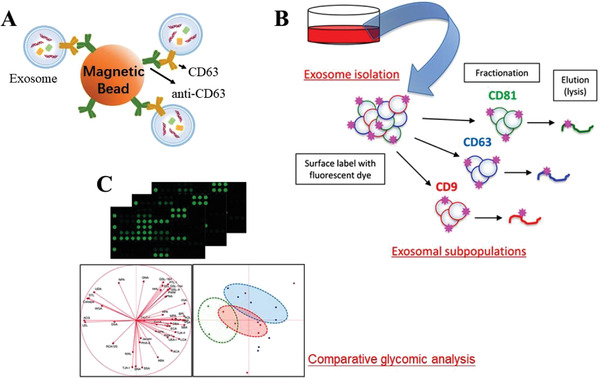
Diagram of exosome separation and application based on immunoaffinity. A) Scheme of total exosome separation by monoclonal antibody‐coated magnetic beads. B,C) Using three types of antibodies (CD81, CD63, CD9) to distinguish exosome‐based on the differences in glycomic expression on surface illustrated by principal component analysis. Reproduced with permission.^[^ ^75^ ^]^ Copyright 2020, American Chemical Society.

The strategies discussed above are based on physical properties of exosomes, like their density, size, and solubility, which may suffer from analog interference or impurity contaminants. To overcome this shortcoming, another simple yet powerful method of exosome separation based on biological immunoaffinity was proposed. They utilized monoclonal antibodies coated magnetic beads to specifically isolate their corresponding antibodies expressed on surface of exosomes.^[^ ^71^ ^]^ These beads can effectively capture target exosomes and allow them for downstream analysis, like flow cytometry, western blotting. Theoretically, any protein or cell membrane component that exists alone or highly on the exosomes’ membrane without a soluble counterpart in the extracellular fluid can be used for the capture of exosomes based on immunoaffinity. Over the past few decades, various exosomes’ markers have been recorded, including lysosomal‐associated membrane protein 2B, transmembrane proteins, heat shock proteins, platelet‐derived growth factor receptors, fusion proteins (such as Lorraine, Annexin, and GTPases), lipid‐related proteins, and phospholipases. Among them, transmembrane proteins such as Rab5, CD81, CD63, CD9, and CD82 have been widely used for selective exosome separation,^[^ ^72^, ^73^ ^]^ and several popular exosome separation products have been produced, including exosome separation and Analysis kit (Abcam), exosome‐human CD63 isolation reagent (Thermo Fisher Scientific) and exosome isolation kit CD81/CD63 (Miltenyi Biotec).^[^ ^74^ ^]^ Due to the ubiquity of transmembrane proteins, the exosomes obtained based on such antibodies are the sum of the various types of exosomes in the biological sample species. The schematic diagram of separation total exosomes was shown in **Figure** **10**A. Matsuda^[^ ^75^ ^]^ applied Thermo Fisher Scientific's CD9, CD63, and CD81 isolation kits to separate exosomes from culture fluid of pancreatic cancer cells, and obtained three different types of exosome solution with corresponding transmembrane proteins (CD9‐, CD63‐, and CD81‐positive exosomes) (Figure 10B). The products were then subjected to lectin matrix analysis and results data were presented by principal component analysis, which showed that the three types of exosomes were successfully distinguished based on the differences in glycomic expression on surface (Figure 10C).

When we try to acquire a type of particular exosome associated with a disease, we should employ specific antibodies to capture them. On the surface of exosomes, a vast array of different cancer‐derived antigens can be detected, such as CEA, EpCAM, HER2, IGFR, LMP1, MUC18, and PSMA. They are highly correlated to the types of host cells and are commonly used as diagnostic and therapeutic indicators for a variety of cancers. The reported exosome biomarkers that have been used are summarized in **Table** **2**. Zhao^[^ ^76^ ^]^ combined three kinds of biomarkers‐conjugated magnetic beads, including CD24, EpCAM, and CA125 antibodies, to differentiate ovarian cancer patients and healthy groups. Results showed that the content of CD24, EpCAM, and CA125 from patients’ blood increased 3, 6.5, and 12.4 times, respectively, which demonstrated the great potential of biological markers in exosome separation and cancer analysis. Besides, Wang^[^ ^27^ ^]^ designed an exosome separation and detection chip. The magnetic beads immobilized with anti‐CD63 antibody were used to capture the exosomes in the urine of prostate cancer patients, and additional Raman signal molecules enables the captured exosomes to display Raman signals and be detected. It is worth noting that, In order to allow the exosomes to fully contact with magnetic beads and increase the capture efficiency, an orderly arrangement of triangular micro‐pillar structure was introduced into the system. The principle of the micromixer is that the fluid flowing through it creates an anisotropic flow in which the substances are constantly mixed and dispersed, eventually resulting in a uniform distribution. The design of this chip provides a reference for improving the efficiency of biological capture of exosomes.

**Table 2:** Markers used for isolation of cancer‐derived exosomes

Type of cancer	Antigen	Ref.
Ovarian cancer	CA19‐9, CA125, CD24, CD171, CLDN3, MUC18	^[^ ^77^, ^78^, ^79^ ^]^
Breast cancer	CD44, CEA, E‐cadherin, EGFR, HER2, IGFR, MIF	^[^ ^80^, ^81^, ^82^, ^83^ ^]^
Prostate cancer	CD41b, E‐cadherin, IGER, PSMA	^[^ ^81^, ^82^ ^]^
Lung cancer	CEA, EGFR, IGFR	^[^ ^81^, ^84^ ^]^
Colon cancer	CD44, EpCAM, FAM1348, IGER	^[^ ^80^, ^85^, ^86^ ^]^
Pancreatic cancer	GPC1, MIF	^[^ ^83^ ^]^

**Figure 11:**
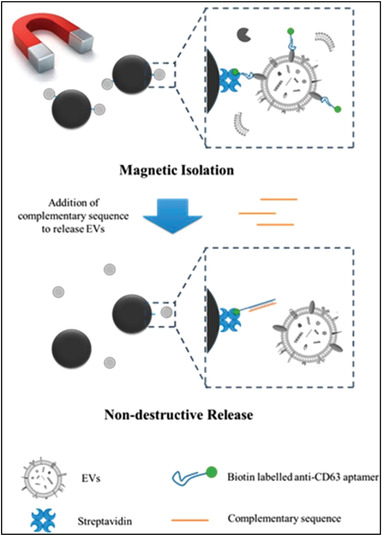
Diagram of exosome separation and application based on aptamer recognition. Aptamer is conjugated on magnetic beads via streptavidin‐biotin linker to capture exosomes. Under the effect of external magnetic field, exosomes‐loaded beads gather up. After that, exosomes can be nondestructively released by addition of competitive complementary sequence of aptamer on account of its structure change. Reproduced with permission.^[^ ^88^ ^]^ Copyright 2019, American Chemical Society.

Although antibody isolation has obvious advantages, its high cost and perishability have hindered their applications, especially in the preparation of large‐scale exosomes. Based on this strategy, another chemically synthesized nucleotide sequence, aptamer, was subsequently and widely employed. On one hand, it can bind to specific target molecules to capture exosomes, such as transmembrane proteins and cancer markers. On the other hand, its synthesis process shows low batch‐to‐batch variation and easy scaling‐up.^–[^ ^87^ ^]^ Typically, aptamer is conjugated on magnetic beads via streptavidin‐biotin linker to capture exosomes (**Figure** **11**).^[^ ^88^ ^]^ Under the effect of external magnetic field, exosomes‐loaded beads gather up. After that, exosomes can be nondestructively released by addition of competitive complementary sequence of aptamer on account of its structure change. The release phenomenon can also be realized by modification buffer system and solution ions (e.g., Mg^2+^ and K^2+^).^[^ ^89^ ^]^ Liu^[^ ^90^ ^]^ reported a thermophoretic aptasensor (TSA) based on the difference in thermophoretic performance of particles with different sizes in the thermal field to capture exosomes. The authors created a thermal field and successfully enriched EVs at high temperatures. These products have been subsequently proven reliable to classify cancer and distinguish malignant and benign diseases. It is also important to note that aptamers need to be stored and used with great care because they are vulnerable to being degraded by enzymes.

### 2.3 Innovative Multi‐strategy for Exosome Separation

When using separation technology based on the principle of immunoaffinity, in order to prevent cells from being recognized and reduce the capture rate of exosomes, biological samples are usually pre‐processed to form a cell‐free sample. According to the aforementioned, these pre‐processing can be realized by particle removal methods such as centrifugation, filtration, and inertial microfluidics. Therefore, in order to efficiently obtain high‐purity products, combining multiple methods to separate exosomes is the future development trend in this field. These innovative strategies are mainly reflected in two aspects. One is to transfer the macroscopic method into the microfluidic system and precisely manipulate particles on the micrometer scale, which can reduce the involvement of large instruments, like ultracentrifuge, and improve the separation efficiency for samples with little volume. The second is to combine multiple strategy to obtain exosome with high purity or specificity for precisely presenting disease information. According to these ideas, some multi‐strategy‐based exosome separation chips were successfully proposed.

**Figure 12:**
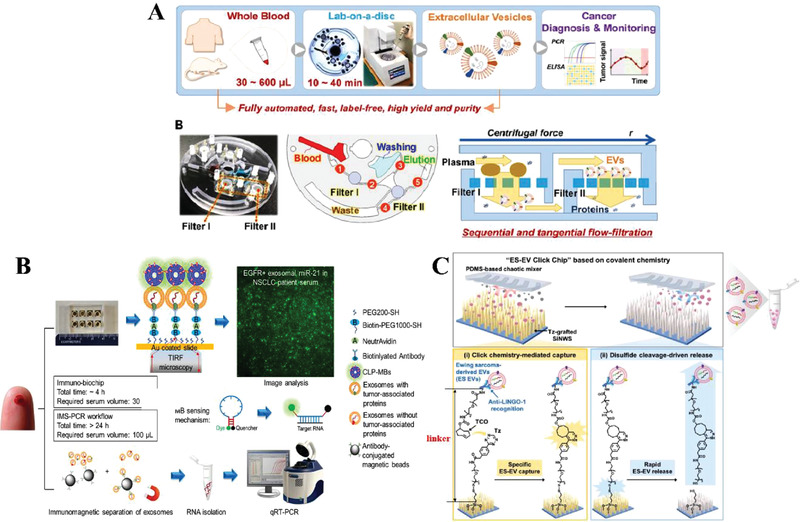
Diagram of multi‐strategy‐based exosome separation on microfluidic chips. A) Diagram of Exodisc separation of exosome. The removal of blood cells and the transfer of liquid are realized by active centrifugation, and the removal of impurities is realized by sequential flow filtration. Reproduced with permission.^[^ ^91^ ^]^ Copyright 2019, Ivyspring International Publisher. B) Diagram of immune‐chip separation of exosomes. Distinctive proteins are conjugated on Au layer by Biotin‐Avidin linker. Cationic lipoplexes containing RNA target‐sensing molecular beacons (CLP‐MBs) allowed exosomal RNA to be detected under TIRF microscopy. On this platform, exosome separation and RNA quantification time are shortened to 4 h, and the sample quantity (30 µL) is reduced to 1/3, compared to conventional magnetic beads separation coupled with PCR determination. Reproduced with permission.^[^ ^92^ ^]^ Copyright 2018, American Chemical Society. C) Diagram of ES‐EV Click Chip separation of EVs. The separation of EV is realized by immunoaffinity effect of a LINGO‐1protein (ES marker) with its counterpart. The subsequent enrichment for vesicles is prepared by break of the linker between anti‐LINGO‐1 and SiNWS (silicon nanowire substrates). The linker between anti‐LINGO‐1 and SiNWS is synthesized by click chemistry using trans‐cyclooctene (TCO) and tetrazine (Tz). Reproduced with permission.^[^ ^93^ ^]^ Copyright 2020, Wiley‐VCH.

For example, an Exodisc device for separating blood exosomes on a fully automatic disk has been reported by combining centrifugation and filtration strategies.^[^ ^91^ ^]^ One blood separation module, two filtering function areas, and three liquid storage chambers (washing liquid, waste, and product) were distributed on the microfluidic chip (**Figure** **12**A). The removal of blood cells and the transfer of liquid were realized by active centrifugation, and the removal of impurities was realized by sequential flow filtration. It should be pointed out that the filtration module used the principle of tangential flow filtration to make the liquid flow direction perpendicular to the membrane plane, which effectively solved the problem of membrane blockage and prevented the appearance of filter cake. This device was used to monitor tumor progression within 13 weeks of live mouse xenograft models. The results showed that the expression of CD9, PSA, PSMA, EGFR, and other proteins increased with the increase of tumor volume. In addition, the isolated exosomes were quantitatively tested for prostate cancer‐specific proteins PSA, PSMA. The data showed that it can effectively distinguish cancer patients from healthy patients. Besides, a system to selectively capture non‐small cell carcinoma (NSCLC)‐derived exosomes on an immune‐biochip was reported.^[^ ^92^ ^]^ Authors fabricated a 15 nm Au layer by electron‐beam evaporation to hold two types of distinctive proteins, anti‐PD‐L1, and anti‐EGFR, with the help of Biotin‐Avidin linker (Figure 12B). Antibodies can specifically capture tumor‐derived exosomes and introduced cationic lipoplexes containing RNA target‐sensing molecular beacons (CLP‐MBs) allowed exosomal RNA to be detected under total internal reflection fluorescence (TIRF) microscopy. On this platform, exosome separation and RNA quantification time were shortened to 4 h, and the sample quantity (30 µL) was reduced to 1/3, compared to conventional magnetic beads separation coupled with PCR determination. Dong^[^ ^93^ ^]^ also developed a novel chip to separate EVs derived from Ewing sarcoma (ES), which was called ES‐EV Click Chip (Figure 12C). On this chip, two support frames were fabricated, a baseplate full of silicon nanowire substrates (SiNWS) for increasing the device surface area, and a serpentine channel using PDMS (polydimethylsiloxane) allowing for more physical contact between nanowires and exosome solution. The isolation of exosomes on this chip was realized by the immunoaffinity effect of a LINGO‐1 protein, especially expressed on ES‐derived vesicles, with its counterpart. Yet, the subsequent enrichment for exosomes was prepared by break of the linker between anti‐LINGO‐1 and SiNWS. The linker was synthesized by click chemistry using trans‐cyclooctene (TCO) and tetrazine (Tz). This study integrated chemical knowledge to develop new pathways of exosome capture and release, and designed different microstructures for better isolation efficiency. The total population of ES‐derived exosomes may be further easily achieved by application of micron filters.

The introduction of microfluidic technology has transferred complex experiments onto micro‐sized chips, making the exosome separation process more precise and controllable, and also conducive to the maintenance of exosomes’ morphology and function. However, the design of microfluidic chips requires substantial efforts to explore the optimal parameters, such as the number of micropillars and channel size (length, width, height). In addition, the fabrication process of microfluidic chips greatly relies on expensive laboratories and extremely sophisticated instruments. Therefore, the development of microfluidic technology for exosome separation requires more in‐depth investigations and multidisciplinary integration of biomedicine and physics.

## 3 Conclusions

Cancer has posed a great threat to human health. The liquid biopsy can facilitate monitoring of cancer prognosis and has played an important role in cancer detection at an earlier stage via a more reliable way. As a new target of liquid biopsy in recent years, cancer‐derived exosomes have been proven to play a critical role in the early diagnosis and post‐treatment monitoring of a variety of cancers. However, due to the small size and complex living environment of exosomes, it is quite challenging to perform high‐efficiency separation and accurate quantitative detection.

The separation of cancer‐derived exosomes has been improved throughout the past decade, and the introduction of microfluidic platforms has greatly shortened the sample size and saved operation time required for exosomes separation, which is suitable for current real‐time detection and future precision medicine. Therefore, the innovative microfluidic approach outperforms the traditional methods. It can be envisioned that it will become a leading research trend to explore deeply the combination of microfluidic technology with multiple separation strategies to develop integrated microsystems for rapid separation of exosomes with high stability and sensitivity, which will ultimately enable a facile and effective operation for the early diagnosis and treatment of cancer.

## Conflict of Interest

The authors declare no conflict of interest.
